# Volume Prediction With Neural Networks

**DOI:** 10.3389/frai.2019.00021

**Published:** 2019-10-09

**Authors:** Daniel Libman, Simi Haber, Mary Schaps

**Affiliations:** Department of Mathematics, Bar-Ilan University, Ramat Gan, Israel

**Keywords:** volume prediction, LSTM, neural networks, change in volume, finance, machine learning

## Abstract

Changes in intraday trading volume are integral to any algorithmic trading strategy. Accordingly, forecasting the change in trading volume is paramount to better understanding the financial markets. This paper introduces a new method to forecast the log change in trading volume, leveraging the power of Long Short Term Memory (LSTM) networks in conjunction with Support Vector Regression (SVR) and Autoregressive (AR) models. We show that LSTM contributes to a more accurate forecast, particularly when constructed as part of a hybrid model with AR. The algorithm is extended to include data about the time of day, helping the model associate the log change in trading volume with the current hour, which yields the best performance of all trials.

## 1. Introduction

In recent years, deep learning became the subject of a growing body of research in many disciplines, including applications in finance (Dixon et al., 2017). Despite its popularity, only a handful of studies have been done on leveraging deep learning methods in volume prediction (Árpád Szűcs, 2017).

As a result of the growth in deep learning applications, neural networks and specifically Long Short Term Memory networks (LSTM) became popular. LSTM networks in particular demonstrated success in natural language processing as well as in predicting the next element in a sequence or even the entire sequence. This ability can also be applied to prediction of financial trends, including change in trading volume of stocks—a subject with high significance as it can be applied to assist in solving a wide variety of financial problems. For example, an algorithmic trader might use the prediction of the trading volume to determine the size of a position on a certain security. Predicting the change in trading volume has applications for risk management, as well. For instance, a trader may decide to limit intraday exposure, e.g., exposure throughout the trading day, in accordance with changes in trading volume. This area of research may also have some applications in regulatory settings. A model that can predict the change in trading volume may be useful in recognizing irregular activity, such as a sharp increase in volume when a decline would be expected.

Despite its importance, thus far, only a limited number of papers have been published on this topic (Árpád Szűcs, 2017). Thus, the prediction of trading volume, and particularly the intraday change in trading volume, is still an open subject with very limited research. This scarcity is even more pronounced when focusing on the use of deep learning methods and specifically LSTM in forecasting, as well as combining LSTM with other algorithms to create hybrid models.

In this contribution, we leveraged the power of LSTM to predict the change in trading volume of S&P 500 ETF (NYSE:SPY) over the course of the trading day. We implemented LSTM on its own as well as a hybrid model where we combined LSTM with other algorithms. Our results show that LSTM contributes to a superior prediction of the change in volume.

We also used a method called Support Vector Regression (SVR), a type of Support Vector Machine (SVM) first introduced in 1995 by Cortes and Vapnik (1995) and more thoroughly explored in Smola and Schölkopf (2004). SVR works similarly to SVM, generating the predictions by finding a hyper-plane that is then used for the regression. As explained further below, we leveraged SVR in conjunction with other algorithms to create several hybrid models. Our goal was to compare the performance of different approaches and discern whether combining such different approaches together yields any improvement over using these same models individually.

## 2. Literature Review

Compared to price, on which plenty has been written, only a small number of articles have been published on predicting volume (Árpád Szűcs, 2017). Still, predicting and generally better understanding volume remains important because many market players and traders are affected by the trading volume. In addition, price and volume are known to be positively correlated, a phenomenon that has been studied at length, particularly during the 1980s by Karpoff (1987). These works focused on finding the long-term correlation between volume and delta price squared, defined as the square of the change in price.

Studies show that the change in intraday trading volume may be affected by a variety of factors, including patterns in the opening, closing, auctions, news releases, and market microstructures, as well as numerous other factors (Kissell, 2014). On the other hand, volume may also be used to predict market volatility, as shown by Fleming et al. (2008), Wagner and Marsh (2004), and Lamoureux and Lastrapes (1990). Thus, forecasting volume is a complex task. This paper seeks to explore the usefulness of LSTM in predicting the change in overall intraday trading volume as well as compare the performance of LSTM in conjunction with other models.

Several recent examples of attempts to predict volume behavior include Alvim et al. (2010) and Chen et al. (2016). In Alvim et al. (2010), the authors tried to predict volume using Partial Least Squares (PLS) and Support Vector Regression (SVR). Both methods outperformed the benchmark, an approach based on the trading volume of the previous time intervals.

A second article is (Chen et al., 2016), where the authors used the Kalman Filter approach in order to predict intraday volume and Volume Weighted Average Price (VWAP), which is calculated by summing the intraday number of shares multiplied by their price and divided by the daily total number of shares. The authors introduced a closed-form expectation-maximization in order to calibrate their model. This forecasting approach outperformed their two benchmarks: (1) Moving Average (MA) and (2) Component Multiplicative Error Model.

While some limited work can be found on the prediction of actual volume and VWAP (Volume Weighted Average Price), papers that attempt to predict the change in volume are extremely rare. One noteworthy article is (Podobnik et al., 2009), where the authors were successful in finding a cross-correlation between the change in trading volume, calculated as log of the daily difference in volume, and the price.

Other than this study, to the best of our knowledge no other work has been published on studying the change in volume. This is surprising, because change in volume can be extremely useful for market makers in their decision-making, especially when dealing with intraday intervals. For instance, certain algorithmic trading strategies might only succeed when trading activity is expected to increase in the next few minutes. For such strategies, long term volume predictions would not be useful. Our research addresses this issue by comparing a few learning algorithms that focus on predicting the next time stamp volume change based on the trading information from a relatively short window of recent activity.

Deep learning started to gain acceptance during the 1980s but recently grew in popularity due to the increase in parallel computation power and availability of massive amounts of data. This led to the development of many types of neural networks, each geared toward solving a different problem. One of these, Recurrent Neural Networks (RNN), were intended for learning on sequential data *x*^1^, *x*^2^…*x*^*n*^ (Goldberg, 2017).

The following formulas explain the structure of the RNN network by showing what is happening in each layer:

(1) $$\begin{array}{lll} R\left(s_{i-1},x_{i}\right) & = & f\left(s_{i-1}U+x_{i}W\right)\\ s_{i} & = & R\left(s_{i-1},x_{i}\right)\\ y_{i} & = & O\left(s_{i}\right) \end{array}$$

Each layer produces two outputs: *s*_*i*_ which is the information passed along the network and *y*_*i*_, which is optional. We can choose a different structure that produces only one output at the last layer. The *s*_*i*_ vector serves as the network memory, which helps the network to keep track of previous inputs when producing the output. The function *f* is a non-linear function such as *tanh*, which is applied element-wise. *W* and *U* are weight matrices that are learned using back propagation.

More recently, we witnessed the rise of Long Short Term Memory (LSTM) networks, which were introduced to address a basic flaw in the ability of RNN to deal with long term memory. LSTM networks are able to handle the vanishing/exploding gradient problem, which was first introduced by Bengio et al. (1994) and further explored in Pascanu et al. (2013). In Hochreiter and Schmidhuber (1997), LSTM networks employ multiplicative gate units to achieve this, adding a memory cell and gate units to the network. The idea is to provide an additional route for historical information to move through the layers without being affected by the vanishing gradient phenomenon. In each layer *t* − 1 the output that passes on to the next layer consists of two vectors: *c*_*t*−1_, which is the memory cell, and *s*_*t*−1_, which is similar to the information that is being passed in regular RNN networks. If we let “⊙” represent entry wise composition, then at layer *t*, the following algorithm is applied:

(2) $$\begin{array}{l} c_{t}=f⊙c_{t-1}+i⊙z \end{array}$$

where $f=\sigma \left(x_{t}W^{xf}+s_{t-1}W^{sf}\right)$ is a gate that is used to control the information that passes from the past by *f ⊙ c*_*t*−1_, which is the information to retain from previous layers. The vector $i=\sigma \left(x_{t}W^{xi}+s_{t-1}W^{si}\right)$ is a gate used to control the new information to add from the vector $z=tanh\left(x_{t}W^{xz}+s_{t-1}W^{sz}\right)$. The new information to add is determined by *i ⊙ z*. The weight matrices *W*^*xf*^,*W*^*sf*^,*W*^*xz*^, and *W*^*sz*^ are all trained using back propagation. However, due to the paths created by the gates, the gradients do not vanish and the long memory can flow through the different layers.

In recent years, there has been a growing body of research claiming to achieve better forecasting results with hybrid models that combine multiple learning algorithms as compared to a single algorithm model. Hybrid models have been successful in financial research applications, as detailed in Cavalcante et al. (2016). One example is (Liang et al., 2009), where the authors predicted future options prices using conventional pricing techniques combined with two learning models: Neural Networks and Support Vector Regression. The authors used this hybrid model on empirical data from the Hong Kong options market and showed that it returned results superior to standard methods used for option pricing. We experimented with hybrid models as well.

## 3. Methodology

For our research, we used minute price and volume trading data of the S&P 500 ETF (NYSE:SPY) between 2012 and 2015. The data was purchased from QuantQuote.

We divided this data into three sections: train, development, and test. The train dataset was from Jan 1, 2012 to December 31, 2013. The development dataset was from Jan 1, 2014 to April 30, 2014. The test dataset was from May 1, 2014 to September 30, 2014. Table 1 below outlines several descriptive statistics metrics on the three different datasets.

**Table 1:** Descriptive statistics on the three datasets.

	**Train**	**Development**	**Test**
Mean	178,135	154,321	119,381
Standard deviation	391,694	285,661	240,427
Median	73,385	58,149	49,015
Number of samples	370,114	63,201	70,005

We trained each of the algorithms described below on the train dataset but selected the best-performing parameters based on the lowest error we got on the development dataset. This was done to achieve cross-validation, since the models are prone to overfitting on the train dataset. We used the parameters to evaluate performance on the test dataset and compared the results of each model. We used the Tensorflow package to build and execute the LSTM algorithm as well as track the results.

We tested a total of nine methods to predict the change in trading volume of the S&P 500 ETF over the course of the trading day. These included LSTM and several other models explained further below.

In order to find the best way to predict change in log volume on a 10-min interval, we experimented with a few methods. The first method, labeled “AR,” was a simple Auto Regressive (AR) model on the log of trading volume figures. We calculated AR using the following formula:

(3) $$\begin{array}{l} {\hat{v}}_{i}=av_{i-1}+b \end{array}$$

where ${\hat{v}}_{i}$ represents the predicted log of the trading volume and *v*_*i*_ represents the log of the actual trading volume. The parameters *a*, *b* are fitted using the intraday volume data, e.g., from the beginning of the training up until the last known value *i* − 1. As the formula illustrates, each prediction is calculated as a linear combination of the last value. Lastly, we generated the prediction of the change in the log volume by calculating ${ŷ}_{i}={\hat{v}}_{i}-v_{i-1}$.

Initially, we fitted the AR model on the log volume of the train dataset. Next, we evaluated the differences, e.g., ŷ_*i*_, on the test dataset. This AR method served as our benchmark.

**Figure 1:**
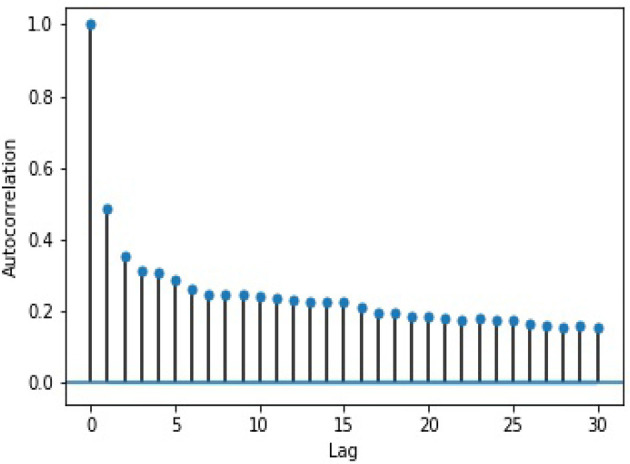
Log volume auto-correlation by lag.

We ran two tests on the log 10-min volume data to ensure that AR is appropriate for our purpose. First, to check whether the data is stationary, we used the Augmented Dickey–Fuller test (Dickey and Fuller, 1979), which returned a result that allowed us to reject the null hypothesis that the data is non-stationary. The test output can be seen in Table 2. From the Next, we performed a lag analysis, which illustrates that the auto-correlation decreases with the lag. The results of this analysis can be seen in Figure 1. Taken together, these provide support for using AR with a lag of 1, or AR(1).

**Table 2:** Augmented Dickey–Fuller test results.

**ADF test statistics**	***P*-value**
−35.462	< 0.001

The second method, labeled “LSTM,” involved running LSTM where the feature vector was comprised of change in log price and log volume over a 50-min window (a sequence of five 10-min intervals). Here, we attempted to predict the change in log volume for the next 10-min interval. Specifically, for each 10-min interval *t* we defined a window *W*_*t*_ as:

(4) $$\begin{array}{lll} W_{t}^{T} & = & (\Delta v_{t-1},\Delta v_{t-2,},\ldots ,\Delta v_{t-5},\\ \; & \; & \Delta h_{t-1},\Delta h_{t-2},\ldots ,\Delta h_{t-5},\\ \; & \; & \Delta l_{t-1},\Delta l_{t-2},\ldots ,\Delta l_{t-5},\\ \; & \; & \Delta c_{t-1},\Delta c_{t-2},\ldots ,\Delta c_{t-5},\\ \; & \; & \Delta o_{t-1},\Delta o_{t-2},\ldots ,\Delta o_{t-5}) \end{array}$$

where Δ*v*_*t*_ is the change in volume, Δ*h*_*t*_ is the change in high price, Δ*l*_*t*_ is the change in low price, Δ*c*_*t*_ is the change in close price, and Δ*o*_*t*_ is the change in open price, all for a 10-min interval. We chose the window size of 5 after some early trial and error suggested that it may have the best prediction potential. However, optimization of the window size along with other model parameters may require additional research.

For our third method, labeled “LSTM-AR,” we added the AR predictions for the log price and log volume into the LSTM feature vector. We accomplished this by calculating the AR prediction set of the log prices and log volumes. The prediction set was comprised of the open, close, high, and low prices during any given 10-min interval. We chose to leverage AR to predict the figures, then calculated the delta between the prediction and the latest actual data. For example, we used AR to predict the next open, then subtracted from it the last known open to arrive at the delta. This was repeated for each 10-min interval in the 50-min window. These delta figures were then incorporated into the LSTM feature vector.

For our fourth method, labeled “LSTM-SVR,” we created a hybrid model combining the results from LSTM with SVR. This was achieved by using the LSTM output as the SVR feature vector.

For our fifth method, labeled “LSTM-AR-SVR,” we used the model from our “LSTM-AR” method, then fed the output into the SVR feature vector.

One of the problems we encountered was that LSTM, by itself, could not capture the U-shape characteristic of the daily volume. This is because the LSTM can only look at a 5-min window, whereas the U-shape typically becomes apparent when examining a longer period of time, spanning several hours or even an entire trading day. In an attempt to help LSTM better understand the daily trends in volume, we decided to add the hour to the feature vector. We implemented this on the “LSTM,” “LSTM-AR,” “LSTM-SVR,” and “LSTM-AR-SVR” models, and labeled them “LSTM-HR,” “LSTM-AR-HR,” “LSTM-SVR-HR,” “LSTM-AR-SVR-HR,” respectively.

The performance of the models were evaluated using three scores: Mean Absolute Error (MAE), Root Mean Square Error (RMSE), and the ability of the model to capture the correct direction of the change (Correct Direction), e.g., whether the next timestamp change of log trading volume was positive or negative. We calculated each metric as shown in Table 3 below, where ŷ_*i*_ represents the predicted log change in the volume, *y*_*i*_ represents the actual log change in the volume and *N* is the number of data points.

**Table 3:** Metric formulas used to evaluate the performance of each model.

**Metric**	**Formula**
MAE	$\frac{1}{N}\underset{i}{\sum }|\hat{y_{i}}-y_{i}|$
RMSE	$\sqrt{\frac{1}{N}\underset{i}{\sum }{\left(\hat{y_{i}}-y_{i}\right)}^{2}}$
Correct direction	$\frac{1}{N}\underset{i}{\sum }[\left\{\begin{array}{cc} 1 & sign\left({ŷ}_{i}\right)=sign\left(y_{i}\right)\\ 0 & else \end{array}\right]$

## 4. Results

**Figure 2:**
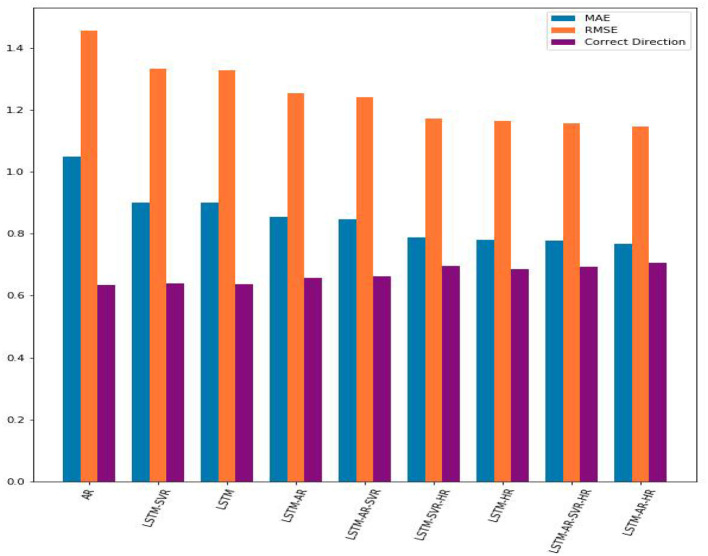
A comparison of the MAE, RMSE, and percentage of correct direction predictions from each trial. The hybrid model, combining LSTM with AR and hourly data, performed the best.

The results of the experiments are shown in Table 4 below, sorted in ascending order by the MAE value, e.g., the best result (lowest MAE) appear in the last row. The results are also displayed in Figure 2.

**Table 4:** Results from each trial run.

	**MAE**	**RMSE**	**Correct direction**
AR	1.0493	1.4570	0.6350
LSTM-SVR	0.9015	1.3331	0.6404
LSTM	0.8993	1.3270	0.6383
LSTM-AR	0.8536	1.2542	0.6571
LSTM-AR-SVR	0.8459	1.2419	0.6620
LSTM-SVR-HR	0.7874	1.1714	0.6954
LSTM-HR	0.7813	1.1646	0.6865
LSTM-AR-SVR-HR	0.7789	1.1560	0.6928
LSTM-AR-HR	0.7669	1.1465	0.7054

As can be seen from the table, LSTM-AR-HR gave the best performance of all models, with MAE of 0.7669 and correct direction of 0.7054. This represents a substantial improvement over the AR results –1.0493 MAE and 0.6350 correct direction. All of the algorithms resulted in an improvement over the AR trial, yielding both lower MAE and higher correct direction.

Interestingly, the LSTM-SVR model produced a slightly lower value of MAE error but performed significantly better in the ability to predict the correct direction of the log change in volume. This can be explained by the SVR's margin, which gives it the ability to understand and learn overall trends in data—in this case, the change in log volume. On the flip side, this also means that the SVR model is less able to capture smaller, more nuanced changes, particularly over shorter time periods.

From these experiments, it is evident that LSTM contributes to a prediction algorithm that is superior to AR. Furthermore, the addition of the hour information into the feature vector further helps LSTM understand and model the data, more so than combining LSTM with other models. However, combining LSTM with SVR and/or AR also improves the model's performance, although SVR is superior to AR when each are combined with LSTM individually. As explained above, the best results are achieved by adding the hour data and combining LSTM with AR.

**Figure 3:**
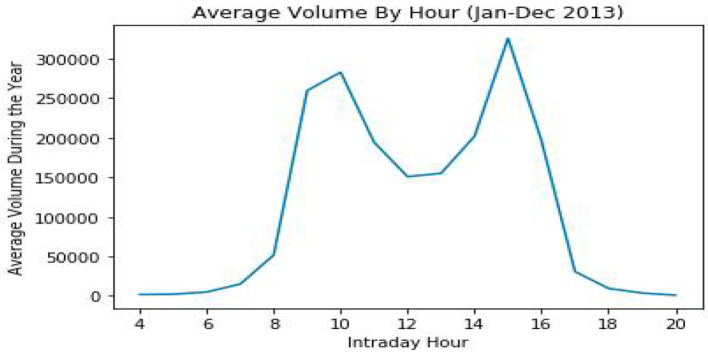
Average trading volumes by hour during 2013. Although daily data can deviate substantially from the average, the typical U-shape is clearly visible, resulting from higher activity in the early and late hours of the trading day along with a dip around mid-day.

Since the hour of the day played an important role in the prediction, we further analyzed its effect and whether it can be used by itself to predict the change in log volume on intraday data. First, the importance of intraday time is evident from Figure 3, which shows the average volume by hour over a 1 year period. In this graph, we can easily notice the U-shape of the average volume, e.g., in certain hours in the day such as mid-day, volume tends to decrease, while at others, such as the early morning and late afternoon, volume tends to increase, on average.

However, attempting to predict the intraday change in log volume based on this phenomenon yields results that are far less accurate than the other methods we deployed. We attempted to predict the change in log volume in a few ways. First, we tried to use the expected average volume, which gave us MAE of 1.1086 and RMSE of 2.256. Next, we used LSTM with a window of five 10-min intervals, where the only feature we sent was the hour—similar to the other algorithms we used in this study. This yielded MAE of 0.9165 and RMSE of 1.3907. In both experiments, our ability to forecast the right direction of the change in the log volume dropped below 60%. This indicates that the time of day, by itself, does not perform well in attempting to predict the change in log volume. In other words, attempting to predict the log change in volume based on whether we would expect the volume to increase or decrease according to the time of day, is not a good strategy. This is because trading data is volatile over individual days. Further, incorporating additional information about actual trading data brings additional information, and leveraging this data along with the power of LSTM is valuable in improving predictions.

**Figure 4:**
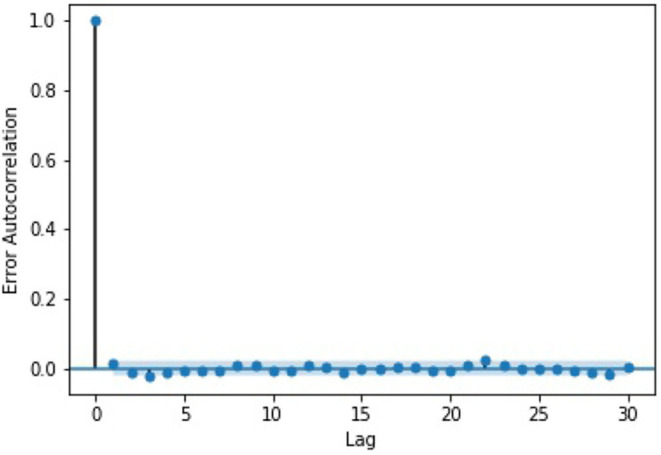
A graph of the error auto-correlation by lag. The thin band represents the 95% confidence interval.

We also examined the errors generated by our best-performing model, “LSTM-AR-HR.” The errors were calculated as the difference between our model's prediction of the change and the actual change in log volume for the interval. For our analysis, we checked for auto-correlations between the errors. The results, as can be seen in Figure 4, show that there is no auto-correlation between the errors in the time series.

## 5. Conclusion

In this paper, our goal was to test the performance of LSTM on its own as well as when combined with other models in predicting the log change in trading volume during the trading day. We compared LSTM, LSTM combined with Supported Vector Regression (SVR), and LSTM combined with SVR and AR, and a combination of all three. We also added the hour into the feature vector, which proved helpful in predicting the log change in volume. We attribute this improvement to the general trend in intraday trading volume, which typically resembles a U-shape with trading volume peaking during the early and late trading hours in a day.

Predicting the change in volume is key in a variety of financial applications, including algorithmic trading, where knowing the change in trading volume can impact the trading strategy. In particular, we focused on predicting the change in trading volume over a short timespan, which is helpful in adopting the most profitable strategy over the next few minutes. Future research can look further into this topic by incorporating additional, newer models to improve predictions. It would also be interesting to explore the variation in the U-shape over the course of different trading days to better understand and perhaps even predict the entire U-shape based on the U-shape of the preceding days, for example.

## Data Availability Statement

The datasets for this manuscript are not publicly available because Purchase required—we purchased stock minute data from QuantQuote. Requests to access the datasets should be directed to daniellibman@gmail.com.

## Author Contributions

As a primary researcher, DL was responsible for collecting and processing the relevant data, writing the code, experimenting with different algorithms, comparing results, and authoring most of the paper. SH provided guidance throughout the project, debating and proposing additional methods to deploy. During the authoring stage, he brought up a multitude of thoughtful comments to help refine the paper and ensure its fit with the institution's academic standards. MS supervised the project. She was instrumental in ideation, direction, and verifying all mathematical calculations. MS also facilitated access to crucial resources, including the data sources as well as several colleagues that served as advisors and mentors throughout the project. She contributed valuable feedback throughout the process that proved essential to obtaining quality results in a timely manner.

### Conflict of Interest

The authors declare that the research was conducted in the absence of any commercial or financial relationships that could be construed as a potential conflict of interest.
